# Supplementary figures and images for: Unveiling the developmental dynamics and functional role of Odorant Receptor Co-receptor (Orco) in Aedes albopictus: A novel mechanism for regulating odorant receptor expression
Source: PLoS Negl Trop Dis. 2025 Nov 26;19(11):e0013753. doi: 10.1371/journal.pntd.0013753 (PMC12680343; doi:10.1371/journal.pntd.0013753)

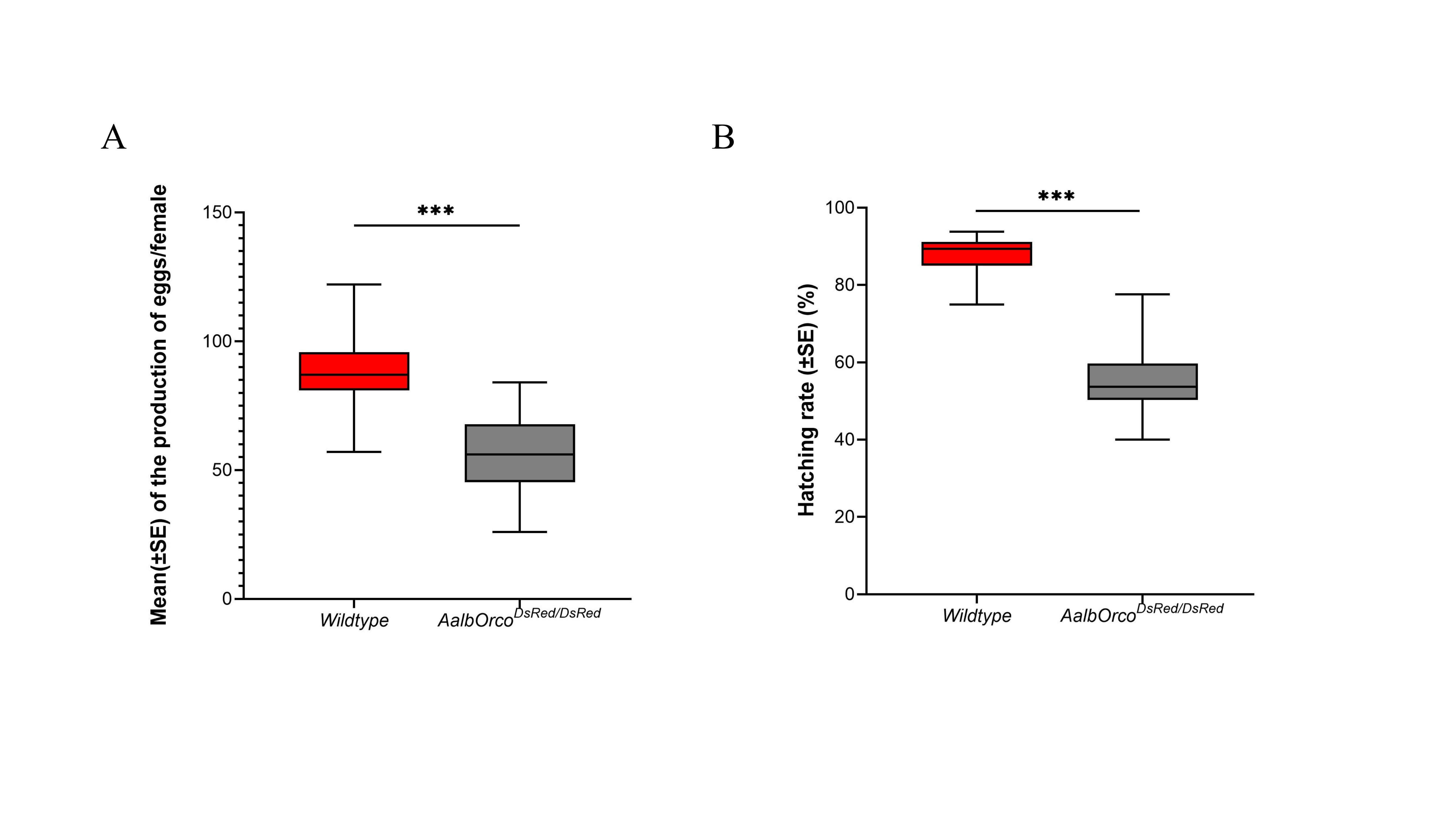

Supplement: S1 Fig — (A) Egg laying of individual female wild-type and AalbOrcoDsRed/DsRed mosquito (n = 8). The number of eggs laid by AalbOrcoDsRed/DsRed was lower than that of the wild-type. (B) Hatching rate of eggs laid by wild-type and AalbOrcoDsRed/DsRed mosquito (n = 8). The hatching rate of eggs laid by AalbOrcoDsRed/DsRed mosquito was lower than that of the wild-type. Mann-Whitney U test was applied in the statistical analysis, statistical significance is presented as P < 0.05 (*), P < 0.01 (**), P < 0.001 (***), and P > 0.05 (ns). (TIF) [file pntd.0013753.s001.tif]

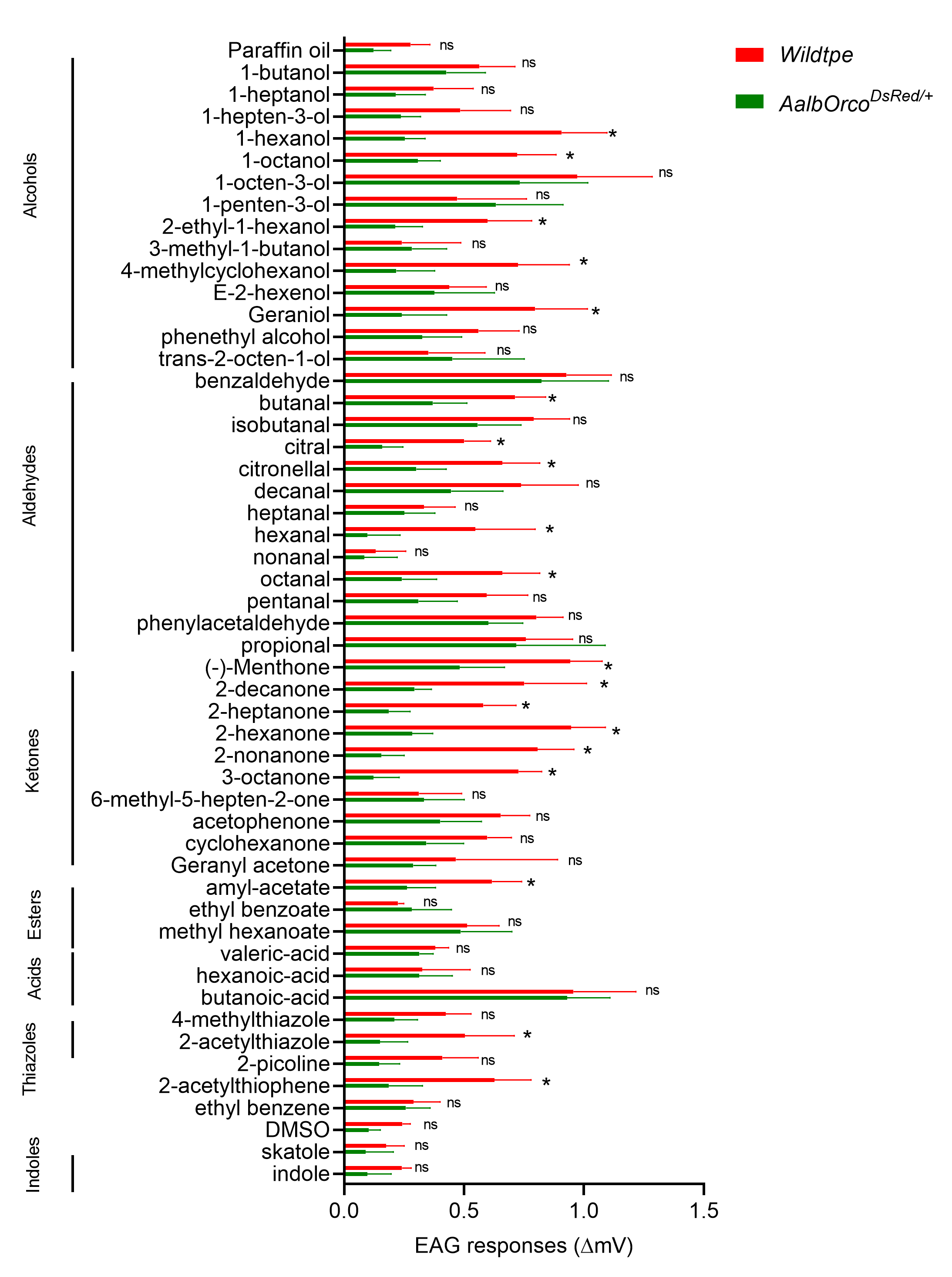

Supplement: S2 Fig — Comparison of EAG responses of wild-type and AalbOrco+/DsRed Ae. albopictus to 50 odorants in different chemical classes (n = 8). EAG responses (∆mV) for each odorant at a 10−1 dilution were normalized to the solvent control (Paraffin oil and DMSO were used as solvents. Indole and skatole were dissolved in DMSO, while the other 48 compounds were dissolved in Paraffin oil.) by subtracting the solvent-induced EAG value. Mann-Whitney U test was applied in the statistical analysis, with P ≥ 0.05 indicating no significance (ns), and P < 0.05 (*) as significant differences. (TIF) [file pntd.0013753.s002.tif]
