# Supplementary material for: Unveiling the developmental dynamics and functional role of Odorant Receptor Co-receptor (Orco) in Aedes albopictus: A novel mechanism for regulating odorant receptor expression
Source: PLoS Negl Trop Dis. 2025 Nov 26;19(11):e0013753. doi: 10.1371/journal.pntd.0013753 (PMC12680343; doi:10.1371/journal.pntd.0013753)
Supplement: S1 Table — Chemical information of odorants, such as CAS number, purity, and company, was included. (PDF) [file pntd.0013753.s003.pdf]

Supplementary Table 1. Odorants lists used in electrophysiological recordings

Chemicals	CAS	Company	Purity
Paraffin oil	8012-95-1	Sigma	100%
1-butanol	71-36-3	Sigma	99.90%
1-heptanol	111-70-6	Sigma	98%
1-hepten-3-ol	4938-52-7	Sigma	98%
1-hexanol	111-27-3	Sigma	98%
1-octanol	111-87-5	Sigma	99%
1-octen-3-ol	3391-86-4	Sigma	98%
1-penten-3-ol	616-25-1	Sigma	99%
2-ethyl-1-hexanol	104-76-7	Sigma	99.60%
3-methyl-1-butanol	123-51-3	Sigma	98%
4-methylcyclohexanol	589-91-3	Sigma	98%
E-2-hexenol	928-95-0	Sigma	96%
geraniol	106-24-1	Sigma	98%
isobutanal	78-84-2	Sigma	99%
phenethyl alcohol	60-12-8	Sigma	99%
trans-2-octen-1-ol	18409-17-1	Sigma	97%
benzaldehyde	100-52-7	Sigma	99.50%
butanal	123-72-8	Sigma	99%
citral	5392-40-5	Sigma	95%
citronellal	106-23-0	Sigma	95%
decanal	112-31-2	Sigma	98%
heptanal	111-71-7	Sigma	95%
hexanal	66-25-1	Sigma	98%
nonanal	124-19-6	Sigma	95%
octanal	124-13-0	Sigma	99%
pentanal	110-62-3	Sigma	97%
phenylacetaldehyde	122-78-1	Sigma	90%
propional	123-38-6	Sigma	97%
(-)-menthone	14073-97-3	Sigma	90%
2-decanone	693-54-9	Sigma	98%
2-heptanone	110-43-0	Sigma	99%
2-hexanone	591-78-6	Sigma	98%
2-nonanone	821-55-6	Sigma	99%
3-octanone	106-68-3	Sigma	98%
6-methyl-5-hepten-2-one	110-93-0	Sigma	98%
acetophenone	98-86-2	Sigma	98%
cyclohexanone	108-94-1	Sigma	99.80%
geranyl acetone	689-67-8	Sigma	97%
amyl-acetate	628-63-7	Sigma	99%
ethyl benzoate	93-58-3	Sigma	99%
methyl hexanoate	106-70-7	Sigma	99%
valeric acid	109-52-4	Macklin	99%

hexanoic-acid	21188-58-9	Sigma	97%
butanoic-acid	107-92-6	Sigma	99%
4-methylthiazole	693-95-8	Sigma	99%
2-acetylthiazole	24295-03-2	Sigma	99%
2-picoline	109-06-8	Sigma	98%
2-acetylthiophene	88-15-3	Sigma	98%
ethyl benzene	100-41-4	Sigma	99%
DMSO	67-68-5	Sigma	99.70%
skatole	83-34-1	Sigma	98%
indole	120-72-9	Sigma	99%
limonene	138-86-3	Sigma	97%
geranyl acetate	105-87-3	Sigma	97%
(+)-fenchone	4695-62-9	Sigma	98%
camphor	76-22-2	Sigma	96%
2-pentanone	107-87-9	Sigma	99.50%
2-methylphenol	95-48-7	Sigma	99%
styrene	100-42-5	Sigma	99%
2,6-dimethylpyrazine	108-50-9	Sigma	98%
4,5-dimethylthiazole	3581-91-7	Sigma	97%
1-pentanol	71-41-0	Sigma	99%
1-hexen-3-ol	4798-44-1	Macklin	98%
methyl acetate	79-20-9	Sigma	99%
butyl acetate	123-86-4	Sigma	99.50%
methyl butylate	623-42-7	Sigma	99%
ethyl-hexanoate	123-66-0	Sigma	99%
