# Supplementary material for: Unveiling the developmental dynamics and functional role of Odorant Receptor Co-receptor (Orco) in Aedes albopictus: A novel mechanism for regulating odorant receptor expression
Source: PLoS Negl Trop Dis. 2025 Nov 26;19(11):e0013753. doi: 10.1371/journal.pntd.0013753 (PMC12680343; doi:10.1371/journal.pntd.0013753)
Supplement: Attachment — Submitted filename: Responses to the comments.docx [file pntd.0013753.s010.docx]

**Methods**

**Reviewer #3:**

**Q1**: Would there be an effect on the experiment when using sucrose water rather than the WHO recommended glucose water for feeding adult mosquitos?
Response 1: Thank you for rising this issue. Our method referred to Wang et al., and based on our observations, feeding adult mosquitoes with sucrose solution did not have significant adverse effects on their survival and reproduction.

**Q2:** Was there ethical clearance for blood feeding assay and host preference assay.
Response 2: Thank you for rising this issue. Yes, our experiment has obtained ethical approval.

**Q3**: Which blood meal source was used to rear the *Aedes* colony at the Shenzhen Bay Laboratory Insectary?

Response 3: Thank you for rising this issue. We feed female mosquitoes with mice.

**Editorial and Data Presentation Modifications**

**Comments 1:** In the methods, I miss the rationale of using mice instead of a human subject for the blood-feeding assay.

Response 1: Thank you for raising this point. The method is described in Wang et al (2017) which used mice for the blood-feeding assays. We use mice blood for mosquito maintenance, and therefore used mice blood for consistency in the assay.

**Comments 2:** Page 3 line 75-78: Rephase

Response 2: Thank you for raising this point. We’ve been rephased these sentences. The rearranged sentences are: “*Dengue fever is one of the most severe tropical diseases transmitted by Aedes mosquitoes with models placing estimates around 390 million cases every year [3] and 4 billion people at risk [4]. Importantly, Ae. albopictus has replaced Ae. aegypti as the primary vector of dengue virus in several countries, such as China and Japan, with its invasive range expanding at a far greater rate than Ae. aegypti due to its superior ability to compete for breeding sites [5,6].*”

**Comments 3:** Page 9 line 360-361: I also miss the rationale of having different starvation times for the blood-feeding (12hrs) and host preference assay (24hrs ) in page 10 line 377.

Response 3: Thank you for raising this point. The starvation time was determined based on the response of the mosquitoes. We attempted to conduct a host preference test using mosquitoes that had been starved for 12 hours, but the mosquitoes were mostly non-responding to the hosts. Therefore, we switched to mosquitoes that had been starved for 24 hours for the test.

**Comments 4:** Page 9 line 342-343: Six libraries were generated. Are the transcriptomes available to the public?
Response 4: Thank you for raising this question. Yes, we will make the transcriptome data public. We have uploaded the transcriptome data of the six libraries to the NCBI Sequence Read Archive (SRA) database, which is currently under the quality review by NCBI.

**Comments 5:** Page 12 line 454: “AalbOrco expression in the labella was exclusively localized to ORNs with…” Do you mean for females or males?
Response 5: Thank you for pointing out this issue. In fact, Aalborco is expressed in T2 sensilla of both female and male labella. Fig. 4 shows the image of female labella.

**Comments 6:** Page 14 line 561: I believe it is not accurate to state that “We observed a significant decline in blood-feeding efficiency in AalbOrco^DsRed/DsRed^ mutants compared to the wild-type mosquitoes”. While the p-value may indicate significance, the double mutants have a feeding efficiency of about 50%, which is more than half of the wild type. There is a relative reduction in feeding efficiency in the double mutants, but these results might also differ if the feeding assay is repeated with human subjects, who are the preferred hosts. Then it would be more relevant from a public health perspective. I believe the data shows feeding success rather than efficiency. A box plot might also present the data better.
Response 6: Thank you for your suggestion. We have revised the description. The results are presented using a box plot, as can be seen in Figure 8B and Figure 8D.

**Comments 7:** Figure 3: The scales have the same numerical value but different lengths.
Response 7: Thank you for pointing this out. These images were captured using objectives with different magnifications, hence the scale bars vary.

**Comments 8:** Figure 6A: Include the results for the diluent.
Response 8: Thank you for your suggestion. The corresponding adjustments can be seen in Figure 6A.

**Comments 9:** In the results and figures indicate whether its female/male or mutant/wild-type mosquito that were used for results presented.
Response 9: Thank you for pointing this out, we’ve checked the description of results and figures and made some modifications.

**Comments 10:** Were there any notable changes in general behavior and probing frequency in the mutants as compared to the controls?
Response 10: Thank you for raising this point. Based on our observation, there is no significant difference in general behaviors between the two strains.

**Comments 11：**Page 5 line 153: I guess you mean lesser-studied species instead of lesser-studies.
Response 11: Thank you for pointing out this issue. We have made modifications,.
**Comments 12：**Page 5 line 181: Can read “7-day-old non-blood-fed females” instead of “7-day-old without blood meal fed..”
Response 12: Thank you for your suggestion. We have made modifications.

**Comments 13:** Page 7 line 272: Do you mean mutant females or wildtype?
Response 13: Thank you for pointing this out. The female mosquitoes mentioned here refer to both mutant and wild-type mosquitoes. We have added this detail in the method description.

**Comments 14:**Page 9 line 343: “three repeats…..” should read “three replicates”
Response14: Thank you for your suggestion. We have made modifications in the transcriptome analysis method.

**Comments 15:** Page 9 line 353: “FPKM” to “FPKM counts”

Response15: Thank you for pointing this out. We have made the modification.

**Summary and General Comments**
**Q1.** However, there are interesting results two Or115 and Or85 over expressed as compared to wildtype in SBTII and their response is significantly high, the speculation is not convincing as Orco role is highly conserved, may be unintended consequence, artifact specifically in that sensilla, may be give a better speculation.

Response 1: Thank you and we completely agree with your comment. Our data could only suggest there is an overexpression of Or115 and Or85. However, the comparable responses between WT and Orco mutants in SBTII are likely due to the presence of other receptor families instead of the overexpression of Or115 and Or85, since Orco is required for the OR responses. The existing results cannot explain why the expression levels of these two genes increase in the mutant. We will continue to conduct further research to uncover the mechanisms.

**Q2.** EAG: how inhibition happens in EAG Fig 6B, is that normalized EAG as response of treatment-solvent?

Response 2: Thank you for pointing out this issue. The responses to the odorants were normalized by subtracting the solvent responses. We consistently observed several odorants elicit smaller responses than the solvent, which is very common based on multiple previous studies in mosquito olfaction.

**Q3.** Fig 8D: Preference index is not clear, feeding(Fig 8B) is fine even though it is reduced, the figure shows preference shitted from human to mouse, I was expecting undifferentiated response to both sides, but now the mutant responded to mouse than human , that is why the response index is negative.
Response 3: Thank you for rising this question. To make the description clearer, we changed the narratives as “*By contrast, host preference towards humans decreased in AalbOrco^DsRed/DsRed^ mutant mosquitoes (P < 0.0001) which instead showed a slight preference to the mice in our study*”. As for the reason why mutants respond to mouse more than human, we speculate that humans have certain key odorants that direct the human preference of *Ae.* *albopictus* (well supported by previous studies in *Ae. aegypti (Zhao et al., Nature, 2022, doi.org/10.1038/s41586-022-04675-4*), however, theses odors may not be sensed by the *Orco* mutants. In addition, the odors that are detected by IRs/GRs might be more abundant in mice, which slightly reverse the mosquito preference.

**Q4**. Did you try how is the response of either physiology or behaviour of heterozygous individuals?
Response 4: Thank you for pointing this out. We conducted additional EAG recording experiments for the heterozygous *AalbOrco^+/DsRed^* mutant and made comparison with the compound-induced EAG responses of the wild-type. The results have been added in the manuscript as supplementary Figure S2.

**Q5.** What is the control for the mutation like parental control?

Response 5: Yes, we used the parental version as a control for the mutant.

**Q6.** Any adverse effect on lifespan, reproduction of the Orco mutation?
Response 6: Thank you for raising this point. The reproductive assays were conducted to demonstrate that the egg production per female and the egg hatching rate are significantly reduced in the Orco mutants (Figure S1).

**Q7.** Line 66-68 ........efficiency, and elimination of host preference in females, there is no elimination they are feeding 50% of them fed(Fig. 8B) but host preference shift is observed but no elimination
Response 7: Thank you for pointing this out. We have made the modification as “*the preference of female mosquitoes for human hosts was significantly decreased.*”

**Q8.** Line 491: While GRs are generally considered absent from antennal expression. Correction: Grs are expressed in a given sensilla found on antenna, need to be rewritten
Response 8: Thank you for pointing out this issue. We have made modifications as “***Previous studies in*** *Drosophila and* ***mosquitoes have identified the expression of certain GRs in the antenna, such as the CO_2_-sensitive GRs in the ab1 sensillum of fly antenna [43] and GR33 in mosquito antenna [44]*” in the manuscript.**

**Minor
Comments** 1: Introduction: too long introduction summarize the story by mentioning the why of the experiment
Response 1: Thank you for rising this question, we have trimmed the content of the introduction.

**Comments 2:** Terminology: Tuning Odorant Receptor Expression, the word tuning should be deleted

Response 2: Thank you for pointing this question, we have made the corresponding modifications.

**Comments 3:** Reviewer #2: (No Response)

**Comments 4:** Reviewer #3: This study delivers a comprehensive spatiotemporal map of Orco expression and function in the mosquito, revealing its dual role as an olfactory co-receptor and transcriptional stabilizer in *Aedes albopictus* and offering new avenues for disrupting host-seeking behavior.

**Comments :** Line 593 "the relatively less complex chemical environment of the aquatic system" This statement would require expounding on the 'less complex'.

Response 5: Thank you for pointing this out. We have made the explanation as “*Compared to several hundred Orco+ neurons in the antenna of adult mosquitoes, the modest number of ORNs (odorant receptor neurons) in the larval antenna may mirror the relatively simplified chemical environment of their aquatic habitat—characterized by a limited diversity of volatile chemical cues and a primary reliance on water-soluble or non-volatile compounds—along with the correspondingly narrower behavioral repertoire required for larval survival (e.g., foraging, habitat navigation, and predator avoidance)*”.
